# Understandings of Participation in Behavioural Research: A Qualitative Study of Gay and Bisexual Men in Scotland

**DOI:** 10.1371/journal.pone.0135001

**Published:** 2015-08-07

**Authors:** Nicola Boydell, Gillian May Fergie, Lisa Margaret McDaid, Shona Hilton

**Affiliations:** MRC/CSO Social and Public Health Sciences Unit, University of Glasgow, Glasgow, United Kingdom; Centre for Geographic Medicine Research Coast, KENYA

## Abstract

An array of empirical research has emerged related to public participation in health research. To date, few studies have explored the particular perspectives of gay and bisexual men taking part in behavioural surveillance research, which includes the donation of saliva swabs to investigate HIV prevalence and rates of undiagnosed HIV. Semi-structured interviews were conducted with twenty-nine gay and bisexual men in Scotland who had participated in a bar-based survey. Thematic analysis of men’s accounts of their motives for participation and their perceptions of not receiving individual feedback on HIV status suggested a shared understanding of participation in research as a means of contributing to ‘community’ efforts to prevent the spread of HIV. Most men expressed sophisticated understandings of the purpose of behavioural research and distinguished between this and individual diagnostic testing. Despite calls for feedback on HIV results broadly, for these men feedback on HIV status was not deemed crucial.

## Introduction

The response of communities of gay men to the emergence of the HIV epidemic have been linked to successful HIV prevention efforts, and the concept of ‘gay community’ has long been considered important in HIV research and sexual health promotion [1–3]. In the context of HIV behavioural surveillance, community-based organisations, venues on the commercial gay scene, and other spaces frequented and used by gay and bisexual men have played a critical role in accessing ‘community’ samples of men from the wider population [4]. Such organisations and venues have also played an important role in HIV education and prevention. It is within this context that the Gay Men's Sexual Health Survey (GMSH Survey) emerged. The GMSH Survey has been conducted every three years since 1996 with gay and bisexual men on the commercial gay scenes of Glasgow and Edinburgh. It was established as part of the evaluation of the Gay Men’s Task Force peer-led sexual health intervention and has gone on to examine changes in gay men's sexual behaviour over time [5, 6]. Since 2005, the survey has included the collection of anonymous oral fluid samples, which are tested for HIV antibodies to assess prevalence of HIV and undiagnosed infection [7]. Undiagnosed HIV infection is determined by comparing self reported HIV status with the results of the HIV antibody test based on the oral fluid sample. Survey data and HIV antibody tests are linked, however, participation in the survey (and provision of a saliva sample) is anonymous [8]. As the biological samples are collected anonymously the men taking part do not obtain any individual feedback on their results.

Research evidence suggests that changes are occurring to the structure of gay communities, patterns of socialisation, and ways of connecting socially and sexually with other men [9–13]. The growing social acceptability of same-sex relationships and homosexuality, have brought about changes to men’s relationships to the gay scene, and sense of connection to gay communities [11–13]. Research in the UK and Australia has identified shifts in societal norms around sexuality, and the increased use of online technologies for engaging with potential partners as facilitators of these changes [4, 14–16]. New technologies such as gay-specific social media, particularly GPS-based apps (e.g. Grindr and Gaydar), have enabled and facilitated changes in social and sexual practices, meaning that in many ways men are less reliant on the geographic places and spaces of commercial gay scenes when connecting with other men [17, 18]. Furthermore, it has been suggested that exclusively gay and/or lesbian venues are reducing in number, in part due to a growing homonormativity, but also the patronisation of gay venues by heterosexual ‘allies’ [17]. As such, there is growing concern that samples of men recruited through convenience sampling and bar-based surveys of gay men, such as the GMSH Survey, may not entirely reflect the diversity of the population, particularly as community norms and practices change [4, 19]. Valid data collection methods are crucial to the development, implementation and evaluation of HIV prevention interventions, and it has been suggested that different methodologies are likely to be required if behavioural surveillance in western countries is to keep pace with changing social and sexual practices [4].

Furthermore, there is a growing ethical question around the acceptability of not providing individual feedback on the results of HIV tests to those who participate in epidemiological surveillance, particularly those with undiagnosed HIV [20–22]. Although much of the research in this area has focused primarily on the use of unlinked anonymous blood samples from participants in low-income countries, issues raised are pertinent to the design of the GMSH Survey. Future behavioural surveillance research could capitalise on developments in HIV testing technologies which allow for immediate results in various settings by including HIV testing within the study design. However, this would require a very different model of data collection than the current bar-based GMSH Survey. Such a change has potential implications for participation; would men be open to this form of survey in this particular context? Although previous research specifically drawing on the GMSH Survey has explored willingness to participate in future HIV prevention studies amongst gay and bisexual men [23] currently little is known about how men understand and perceive the issue of feedback on anonymous HIV antibody tests.

Understanding why people participate in research, and their expectations of feedback, is important in terms of the design and development of effective research strategies. A number of qualitative studies have been conducted which explore the meanings people ascribe to participation in various types of health research. Such research has been conducted with a range of groups including children [24], pregnant women and mothers[25], individuals who have been involved in nursing research on emotive topics [26], and those who have taken, or are considering taking, part in longitudinal cohort studies [27, 28]. Much of this research emphasises people’s perceptions of taking part in studies as an altruistic act, as a selfless contribution to medical science for the wider benefit of society [27, 29–32]. Other key considerations around taking part in research include the potential for improvements in participants’ own health [33]. Some studies report more proximal motivations for research participation, specifically personal benefit. For example, Hallowell and colleagues’ [34] study exploring participation in genetics-related research emphasises the complex interrelation between perceived individual, familial and social benefits. They argue that it is overly simplistic to frame research participation as altruistic, and note the importance of social and familial context in shaping motivations for research participation. A similar theme emerges in the work of Sikweyiya and Jewkes [35]. In their study exploring participation in research around gender based violence in South Africa, they found that self interest and altruism can intersect. Participants in their study often sought to balance individual benefits (and risks) with potential benefits to others.

Some studies also included the perspectives of participants, like many who took part in the GMSH Survey, who had donated tissue samples [29, 30]. Dixon-woods and Tarrant [30] explored people’s perceptions of participation in research, drawing on accounts from individuals from three separate research projects, one of which involved the donation of a tissue sample. The findings of their qualitative analysis suggest that people’s participation in research is dependent on their perception of researchers’ commitment to mutual cooperation. In particular, participants assessed institutional factors and sought indicators that they were engaging with bona fide researchers who were conscientious about protecting the interests of those who contribute to their studies, thereby emphasising trust in researcher integrity. It is important to note that research exploring the issue of tissue sample donation has not previously been conducted with gay men.

Understanding factors which motivate gay and bisexual men to participate in behavioural research, and their perceptions of feedback on anonymous HIV antibody tests can provide valuable insights which could be used to inform the design of future studies. In order to develop our understanding of men’s perspectives on participation in research we developed a qualitative study to explore why men participate in the GMSH Survey.

## Methods

Semi-structured telephone interviews were conducted between October 2011 and December 2011 with 29 men who had participated in the GMSH Survey. The College of Social Science Research Ethics Committee at the University of Glasgow reviewed and approved the study and consent procedure [Application no. 2011044].

Men were recruited in 13 licensed premises in Edinburgh (seven bars) and Glasgow (six bars). Following the GMSH Survey approach to recruitment [23], bars were visited at two different time points; in the early (7:00–9:00PM) and late (9:00–11:00PM) evening. Fieldwork staff (team of five with one team lead) spent half an hour in each venue and approached all men present to explain the nature of the study and invite them to participate. Men were asked if they took part in the GMSH Survey in 2011, if they provided an oral fluid sample during the survey, and if they were willing to take part in a telephone interview as part of a qualitative study exploring men’s perceptions of sexual health research. At this point participants were asked to provide written consent to be interviewed, as well as their first name and a contact telephone number. The fieldworker who recruited the participant then contacted the participant at an agreed date/time to conduct a telephone interview. Participants provided written consent to be interviewed at the time of recruitment, and consent was re-confirmed verbally (and audio recorded) at the start of the telephone interview. In total, 267 men were approached, of which 196 men were eligible to participate in the study. Of the men eligible to participate, 88 men were recruited to the study, and 46 men completed a telephone interview. For the purposes of this analysis, we explore the accounts of 29 men who participated in the 2011 GMSH Survey (S1 Table), the other 17 participants were excluded because they had not. Of these men, 19 had completed the GMSH questionnaire, and 10 had both completed the GMSH questionnaire and provided a saliva sample as part of the survey. Participants ranged in age from 18 to 61. No further demographic data, other than age, were collected as part of the study recruitment process. During the interviews participants were asked about their motivations for participating in the GMSH Survey and their views on the current study protocol which does not provide men with the results of their individual HIV tests. Interviews lasted approximately 30 minutes and were digitally recorded, transcribed verbatim and checked and anonymised.

To enable systematic comparisons to be made across the data, transcripts were imported into NVivo 9. The data were thematically coded and systemically charted, following the principles of framework analysis [36]. All authors contributed to the preparation of a coding framework, which was systematically applied to each transcript. Coding was regularly reviewed by the research team to identify and incorporate emergent themes. The data were then examined by Author A and Author B to identify typical quotes and common reasoning [37]. Throughout the analysis particular attention was paid to contradictory cases. Here, we present findings on i) men’s motivations for participation in the GMSH Survey, and ii) their views on HIV testing as part of the survey.

## Results

### Perceptions of and motivations for participation

A key theme across the men’s accounts was the idea of participation in sexual health research as a means of benefitting wider society. The desire to contribute to ‘the greater good’ was an overarching motivation for participating in the GMSH Survey; for the most part participation was not framed in terms of individual benefit, rather, as a way of helping others. For example, Edgar commented:

I would always tend to take part in these things if asked to for, inverted commas, ‘greater good’. It’s just to aid improvement in health, generally. (Edgar, 45+, survey only)

Similarly, Kiram noted:

If research can help other people, then if I can help by being part of it, then I don’t see why not […] If this research, in the long run, helps other people, then… Then it’s not costing me anything to take part in the research. (Kiram, 45+, survey only)

A number of men suggested that participation in the GMSH Survey was a responsible action, contributing to wider social and public ‘good ‘: “I just think it’s the responsible thing to do, you know, in terms of collating information” (Derek, declined to give age, survey and sample).

Research in general was perceived as a necessary part of advancing ‘science’, and a refusal to participate in the GMSH Survey (and other heath research) framed as potentially regressive. As Roland noted, “If people said no to all this research, we would still have a square wheel, wouldn’t we?” (Roland, 45+, survey and sample).

The GMSH Survey was often framed as contributing to the development of further knowledge about ‘health issues’, specifically the gathering of accurate data on sexual behaviour and the prevalence of HIV (and other STIS). Men emphasised the need to gather such data, and were keen to contribute not only on an individual level but also as a way of facilitating the collection of population- or community- level data. As David explained:

For myself, it’s just contributing you know, in some sort of small way to an accurate picture of how we stand at the moment as a sort of like, within a sort of like minority section of the larger community in society. (David, 25–34, survey and sample)

Similarly, Derek noted:

And to get a good cross-section of how, kind of, general health is within the gay community–so I just thought it’d be a useful thing to do, to contribute to the data capture. (Derek, declined to give age, survey and sample)

Although men framed participation in research as a way of contributing to the greater good, overwhelmingly men emphasised participation in the GMSH Survey as a way of benefiting the wider “gay community” (Tomas, 23, survey and sample). For example, when asked about what motivated him to take part in the GMSH Survey, Taylor explained:

I feel it’s important that these surveys are carried out, and that it’s important for me as well to take part in them. I would say I would actually, sort of, giving something back to the gay community by taking part in things like that. It might seem daft, but that’s my personal opinion. I think it’s a good thing to take part in these things. That way that these, they really need these, research isn’t for nothing. (Taylor, 45+, survey and sample)

Elaborating on this issue, Hamish explained why he chose to part:

Possibly knowing that some benefit was going to come to HIV prevention in the gay community. […] I just think, again, it’s just going back to it being beneficial and, you know, of importance to the gay community. (Hamish, 35–44, survey and sample)

A number of men stressed the value of findings from the GMSH Survey, noting that these could be used to inform the development of health policies aimed at improving the sexual health of gay and bisexual men: “Just so that things like, well, health campaigns can be tailored towards me and other people like me” (Hamish, 35–44, survey and sample). In this way, participation in the survey was framed as a means of contributing to research evidence which could be used to target HIV prevention efforts. Hamish’s comments also reflect how some men perceived participation as having a personal benefit to them as an individual within the wider community.

The men’s accounts suggest that to some degree convenience played a role in decision-making around participation in the GMSH Survey. A common response to questions about participation in the survey was that taking part required little commitment, and caused minimal inconvenience: “…if it helps towards research, it’s no skin off my nose, if you know what I mean.” (Norman, 45+, survey and sample). Furthermore, being approached while socialising in venues on the commercial gay scene meant that the men were not required to be pro-active, appearing to make the decision to participation somewhat easier. As Roger noted:

The reasons for taking part, it just, you know, it was, how can I say–it was not proactive, you know? The researchers were there and they looked quite friendly and the bar was not too busy, so I thought, you know, I could answer to the questions, privately–so yeah, that was it. (Roger, 35–44, survey only)

Similarly, Cameron noted:

I think it was just a case of the folk were there, and obviously they asked, basically. There wasn’t much more–I didn’t actually give it a whole lot of thought at the time. I was there with my mate, and they approached and asked, “Would you mind filling this out?” I’m like, “No problem,” you know? It’s one of these things, obviously you want the information gathered, so I was happy to do it. (Cameron, 25–34, survey only)

The men’s accounts suggest that the relatively small individual ‘cost’ of participating was often weighed up against the potential benefits to wider society, more specifically to ‘men like me’; an ‘imagined community’ of gay men. However, it is worth noting that a number of men commented that other forms of research requiring a large time commitment could be off-putting. Nevertheless, men’s accounts suggest that some would be willing to participate in longitudinal or clinical research if they deemed the benefits to themselves and others to be sufficiently important.

The perceived legitimacy of the organisation undertaking research also appeared to play an important role in men’s decision-making around participation in research. Aligned with their desire to contribute to the ‘greater good’, many men stressed a concern with taking part in worthwhile research, conducted by well recognised and respected organisations. The notion of ‘good’ or ‘legitimate’ research was contrasted with that conducted by ‘corporate’ organisations, specifically organisations which were perceived as using data/information collected to generate profits. Where research was understood as contributing to commercial gain, men expressed concern that this did not align with their conception of research as benefitting others. This prompted some to suggest that they would be less likely to participate in such research. For example, Ollie commented:

If I didn’t feel it was being conducted by an appropriate organisation, the manner and amount of information given at the start of the survey, whether or not it was considered a professional survey that was going to kind of enhance, not enhance… What the purpose of the survey was for and what the results were going to be, if it was just, I can’t think–a drugs company doing it for some gain which would result in, maybe, increased profits, then maybe not, but if it’s for the general health and for, maybe, a non-profit organisation. (Ollie, 25–34, survey and sample)

The legitimacy of the research (and the researchers) was/were also linked to issues of confidentiality and anonymity. In general men perceived researchers employed by ‘legitimate’ organisations as adhering to high ethical standards. In relation to the GMSH Survey, a number of men made specific mention that they trusted the researchers undertaking the survey to act in way that served the best interests of survey participants. This was reflected in the assumption that the research team would uphold/safeguard the anonymity and confidentiality of participants. As Ross explained:

I just think that there’s a good ethos about these organisations that participate in these things, that I don’t have any problem with being anonymous or anything like that, because I know that my research results are confidential, or whatever information you take from it and whatever information I can get from it is just beneficial. (Ross, 18–24, survey and sample)

A small number of participants specifically referenced their knowledge of the organisation funding and undertaking the research, noting that they were well known and respected, and stressing that this gave them confidence in the research. Indeed, that the GMSH Survey was widely recognised as long-running survey, conducted by a well-respected organisation, and being of benefit to the sexual health of wider communities of gay men, appeared to inform men decision-making around participation.

### Perceptions of feeding back HIV results

Aligned with the dominant perception of taking part in the GMSH Survey as a means of making a positive contribution to the ‘community’, most men suggested their donation of a saliva sample was a key element of participation and not contingent on receiving individual HIV test results. For instance, when asked about his perceptions of not receiving individual feedback, Roland commented:

I never really gave it much thought. I just thought, you know, it’s part of this study, or whatnot, that we’re doing. It wasn’t, you weren’t being tested, you know? You were just like a test subject, really. You’re not being tested personally […] So I just thought, you’re just part of a bigger, you know, a bigger survey, really–so I didn’t really think about it like that. (Roland, 35–44, survey and sample)

The men stressed that participation in the survey and the provision of a saliva sample were ways of contributing to understandings of the prevalence of HIV: “I didn’t see it as being a test for my benefit. It was, I think, probably more to gain an idea of numbers, or you know, a percentage of positive or negative people” (Kennedy, 35–44, survey and sample). Indeed, many men elaborated that they viewed HIV testing as an individual’s responsibility and not the purpose of the GMSH Survey.

I didn’t do it as an HIV test, I just did it for a piece of research. I know I can go up to a [sexual health clinic] and get tested for HIV and get my results that way, you know? I wouldn’t offer it as a HIV test, per se, it was just part of research. So you know, for me, it didn’t really come into the equation about results, you know? That wasn’t the purpose of it, really […] if I wanted HIV results or tests, I’d have gone up to the [sexual health clinic]. (Ollie, 25–34, survey and sample)

Similarly, Keith commented:

It’s really your responsibility to be checked up, anyway, by your doctor, or by a centre that deals with it. I mean, I don’t think the key is to tell you what sort of diseases you may or may not have–I don’t think that’s the point to the survey. I just thought it was just generally based around the kind of bigger picture. It’s not an individual thing–I think it’s more a group, team thing, really, to be honest with you. (Keith, 18–24, survey and sample)

In their accounts of decision-making around participation most of the men conveyed a distinction between participation in research and their perceptions of HIV testing. This was in conjunction with their perceptions of the survey as a means of estimating HIV prevalence within a community sample of gay and bisexual men drawn from Glasgow and Edinburgh, and more clinical settings as the appropriate environment for testing individual HIV status.

Some men also suggested that not receiving the results of the HIV test was indicative of the guaranteed anonymity of participation in the survey. For instance David commented:

I just considered it [the saliva sample] as part of the survey, that it would be just completely anonymous. My saliva test wouldn’t have any connection to my name or anything like that and it would just be a number so I didn’t expect or wonder why I wouldn’t get individual results because I didn’t think it would be linked to me. (David, 25–34, survey and sample)

Men’s understandings of the lack of feedback about HIV test results as safeguarding participants’ anonymity relates to participants’ primary considerations about the legitimacy of the research, a key issue in deciding whether or not to take part in survey research.

Some men also suggested that receiving the results of the HIV test would negatively impact their willingness to participate in the GMSH Survey:

I would be more disinclined to participate in a survey and provide a sample [if results were provided]. I think […] it wouldn’t be a general kind of like ‘in the bar’ survey. It would have to be something that I would be booked in for and general kind of geared up for, because at any time that I’ve ever had HIV tests or any other STI test, I know my own mind and I know that I would just fall to bits if I got anything, so that’s why I would require you know, the structure and the sort of like support rather than just sort of like doing it over the phone or that sort of thing. I know what I need rather than just being told something or… I prefer to have sort of like, this is what happens, here’s who you get in touch with, here’s your results. Here’s where we go from here, that sort of thing and having the confidence of that being available. (David, 25–34, survey and sample)

In accordance with many men’s perceptions of a clear distinction between HIV testing for research purposes and for individual results, David and a small number of other men suggested that the prospect of receiving results altered their understandings of participation in the survey. Receiving results blurred boundaries between participating in worthwhile research and undertaking a HIV test in an inappropriate environment where supportive resources were not provided.

In contrast, despite articulating sophisticated understandings of the purpose of the saliva sample as a means of estimating HIV prevalence rather than individual diagnostic testing, many men suggested that feeding back results of HIV tests on donated saliva samples would be generally well-received. A number of the men interviewed, after justifying why results were not routinely fed back, commented: “I suppose it might be handy if you did get the results” (Hamish, 35–44, survey and sample), and “It would have been nice to have got them, but then, you know, it’s just one of those things” (Tomas, 18–24, survey and sample). Some also suggested that getting results could be advantageous for both individuals and the wider ‘community’:

The advantage would be that, you know, if you had a positive result, then you could take steps to deal with that. That’s probably the biggest advantage, I would think […] I’d say there’s a lot of people who don’t know they’re HIV positive, so you know, there’s probably a lot of undetected HIV. (Roland, 35–44, survey and sample)

Some men went on to suggests that by feeding back the detection of an individual’s previously unknown HIV positive status the research team could prompt individuals to seek treatment early, reduce undiagnosed infection amongst gay and bisexual men, and prevent onward transmission of the disease.

Only a small number of men expressed concern about not receiving results from the HIV test performed on the sample they donated. These responses reflected some men’s understandings of the researchers as having a moral responsibility to feedback results to men. For instance Cameron commented:

I certainly think, if you’re asking someone to provide such a thing for that purpose, […] it’s something that could play on someone’s mind after the event, and it’s almost that thing, like, them not knowing and someone else potentially knowing what kind of result they could potentially have. So I definitely think that should be something that’s actually offered, rather than not. (Cameron, 25–34, survey only)

Harry further elaborated:

I would say they must [provide feedback], they should try and be a way to come back to somebody. I mean, at the end of the day, it’s all about prevention, and if somebody could be carrying something they’re not aware of. […] I mean, a contact number relates to a number, I don’t know that you can come back to somebody and say, “Look, there’s a problem here. You need to go and get yourself assessed again.” I think that could be advantageous to a few people and would help prevent the spread. (Harry, declined to give age, survey only)

This response suggests Harry’s expectation of a shared understanding of the importance of minimising HIV transmission—“it’s all about prevention”. Rather than aligning with most men’s articulation of a clear rationale for not providing individual results, these men found the prospect troubling. For these men, lack of feedback on the results of the saliva sample was perceived as not being in the best interests of individual participants and running counter to broader HIV prevention efforts.

Despite varied views on whether or not men should receive individual results, many of the men suggested that in the event of results being fed back, the environment and delivery of this feedback would need to be carefully considered. For instance Ollie commented:

I suppose if they’re going to give results, it depends how the results are given, it’s probably, they would probably need to be given by a qualified person who’s kind of got the training to give such results, just in case they were positive. And also, again, the environment where the results would be given, what type of back-up would be there in case they weren’t quite as you expected. (Ollie, 25–34, survey and sample)

A number of the men suggested that results should be fed back by medical professionals or those who were trained in counselling, rather than researchers. They suggested feedback should be sensitively delivered and in general should be done face-to-face, particularly where the result was positive.

## Discussion

Broadly, our findings are in accordance with previous studies which suggest that people participate in research primarily for altruistic reasons [29–32]. The importance of contributing to ‘the greater good’ was emphasised throughout accounts, in particular the men conveyed a willingness to contribute to research as a means of furthering knowledge for health improvement. The findings of this study, also suggest, similar to Peel et al’s [31] findings, that participation in research is contingent on its convenience. The men in this study suggested that their participation involved little risk to themselves or investment of resources and therefore was ‘no skin off my nose’.

Although the participants in this study alluded to broadly altruistic motives and the convenience of participation, they defined their motives for participation primarily by the specific context of the GMSH Survey. Their ‘contribution’ was seen as being particularly important to an ‘imagined community’ [38, 39] of gay men. Benedict Anderson [38] conceptualised ‘imagined community’ as community extending beyond local, face-to-face ties, to include those that are ‘imagined’ to belong to a community. This is particularly useful in conceptualising gay communities as not all ties may be face-to-face, rather, may be engendered by a *sense of belonging* to a wider community of gay men. Indeed, the men’s emphasis on the importance of the GMSH Survey to health improvement for this ‘imagined community’ of gay men was cited as a key motive for participation. In line with the work of Hallowell et al [34] and Sikweyiya et al [35] this serves to emphasise the importance of social context in shaping motivations for participating in research.

A review of literature examining barriers and facilitators to research participation among minority ethnic groups in the United States [40] found that community ‘buy-in’ was particularly important to target populations whose participation was contingent on research being aligned with ‘community priorities’. The findings of this study similarly highlight the continued importance of buy-in from the population being targeted as a means of ensuring participation. Nevertheless, given the findings of this study, it is interesting to consider how shifts in men’s sense of belonging to gay communities may impact attitudes to research. Research has found that some gay men, particularly young men, may be ambivalent about the concept of gay communities [13, 41, 42]. Further research could explore how changes to men's relationships with and to gay communities may affect participation in behavioural research. Indeed, whatever form future behavioural surveillance research takes, the continued support and buy-in by gay men, and the gay communities they imagine themselves to be part of, continues to be vital to the ongoing success of future research efforts.

Our findings also align with wider assertions about the importance of gay community leadership in HIV prevention, as leaders and active stakeholders in the development of much HIV education, research and advocacy [3]. Men’s understandings of their participation in research as one of the ways they ‘make a contribution’ to HIV prevention, and health promotion, policy and practice in relation to gay men’s health reinforces the need for continued public engagement and dissemination of findings in a way which demonstrates to participants how they have contributed to relevant policy, practice and the development of future research.

The findings of our study also offer an alternative perspective on the importance of providing individual feedback on the results of HIV tests to those who participate in epidemiological surveillance, particularly those with undiagnosed HIV. Although increasingly recommendations suggest the importance of providing this feedback [20–22], our study highlighted how men’s understanding of the purpose of their participation as a means of contributing to behavioural surveillance research was distinct from their expectations of HIV testing, where they would expect feedback on HIV status. Indeed, most men viewed the relatively unobtrusive act of providing a saliva swab for HIV testing as a crucial part of the research and not a means of HIV testing. However, perhaps if they had provided a blood sample their expectations of feedback would have been more complex [43]. While ethical considerations continue to shape the design and development of studies, this study suggests that where participants’ understanding is well-developed and their consent informed by broader knowledge around the subject and purpose of research individual feedback on results may not always be necessary, particularly where testing is widely and freely available elsewhere.

A key limitation of the study was that we were unable to recruit men who did not participate in the 2011 GMSH Survey. The original research design included the collection of data from men who had declined to take part in the GMSH Survey. The aim of this was to explore factors that acted as barriers to participation. Seventeen men who had not taken part in the GMSH Survey were recruited to the study, however, during interviews it became clear that these men had not refused to take part, rather had not been approached during the 2011 study period. All 17 of the men explained that had they been approached, they would all have agreed to participate. Inclusion of men who refused to participate would have added robustness to the study. Further limitations of our study include the context in which the men were recruited to the study. Similar to previous research with men recruited to the GMSH Survey, exploring willingness to participate in future research [23], the sample was limited to gay men who were recruited on the commercial gay scene. It is perhaps not surprising that ‘community’ narratives are particularly prominent in this setting, due to of the long history of scene-based health promotion and health advocacy in urban areas of Scotland. The findings of the study are perhaps not more broadly relevant to groups of gay and bisexual men who do not frequent such venues. Given recent suggestions around the changing structure of gay communities, patterns of socialisation, and approaches to meeting other men [9–13], those men who do not frequent the commercial gay scene might have different views on participation as well as alternative understandings of the purpose of research.

## Conclusion

Continuing to engage with gay and bisexual men, and practitioners working within these communities, as part of the process of developing new and innovative research strategies is critical to engendering support for, and trust in, future behavioural research. This underlines the need for continued engagement with stakeholder groups, both during the development phase of research studies, but also, in dissemination of the research findings. Further research is also needed to explore men’s perceptions of participation in research, and their perspectives on receiving feedback on testing, within wider contexts. Specifically, exploring if and how men’s social and sexual interactions with other men impact their understandings of research participation. For instance research could usefully explore the perspectives of those men, not visible on the commercial gay scene, who could perhaps be recruited through social media networks [18]. Indeed, it is crucial to continue to explore the opportunities and limitations of particular recruitment strategies to ensure they capture the heterogeneity of the population or ‘community’ they seek to engage.

## Supporting Information

S1 Table Participant Characteristics. (PDF)

## References

[1] Kippax S, Connell RW, Dowsett GW, Crawford J. Sustaining safe sex: Gay communities respond to AIDS London: The Falmer Press; 1993.

[2] Kippax S, Crawford J, Connell B, Dowsett G, Watson L, Rodden P, et al The importance of gay community in the prevention of HIV transmission: A study of Australian men who have sex with men AIDS: Rights, risk and reason: The Falmer Press London; 1992 p. 85–101.

[3] Trapence G, Collins C, Avrett S, Carr R, Sanchez H, Ayala G, et al From personal survival to public health: community leadership by men who have sex with men in the response to HIV. Lancet. 2012;380(9839):400–10. 10.1016/s0140-6736(12)60834-4 . 22819662 PMC3805044

[4] Zablotska I, Holt M, Prestage G. Changes in gay men’s participation in gay community life: Implications for HIV surveillance and research. AIDS Behav. 2012;16(3):669–75. 10.1007/s10461-011-9919-9 21424273

[5] Williamson LM, Hart GJ, Flowers P, Frankis JS, Der GJ. The Gay Men's Task Force: The impact of peer education on the sexual health behaviour of homosexual men in Glasgow. Sexually Transmitted Infections. 2001;77(6):427–32. 10.1136/sti.77.6.427 11714941 PMC1744413

[6] Knussen C, Flowers P, McDaid LM, Hart GJ. HIV-related sexual risk behaviour between 1996 and 2008, according to age, among men who have sex with men (Scotland). Sexually Transmitted Infections. 2011;87(3):257–9. 10.1136/sti.2010.045047 21071563 PMC3272888

[7] McDaid LM, Hart GJ. Increased HIV testing and reduced undiagnosed infection among gay men in Scotland, 2005–8: Support for the opt-out testing policy? Sexually Transmitted Infections. 2011;87(3):221–4. 10.1136/sti.2010.044560 21325443 PMC3791475

[8] Williamson LM, Hart GJ. HIV prevalence and undiagnosed infection among a community sample of gay men in Scotland. JAIDS Journal of Acquired Immune Deficiency Syndromes. 2007;45(2):224–30. 10.1097/QAI.0b013e318058a01e . 17417102

[9] Rosser BRS, West W, Weinmeyer R. Are gay communities dying or just in transition? Results from an international consultation examining possible structural change in gay communities. AIDS Care. 2008;20(5):588–95. 10.1080/09540120701867156 . 18484330 PMC2562784

[10] Davis M. The ‘loss of community’ and other problems for sexual citizenship in recent HIV prevention. Sociology of Health & Illness. 2008;30(2):182–96. 10.1111/j.1467-9566.2007.01050.x 18290931

[11] Reynolds R. What happened to gay life? Sydney: UNSW Press; 2007.

[12] Reynolds R. Endangered territory, endangered identity: Oxford Street and the dissipation of gay life. Journal of Australian Studies. 2009;33(1):79–92. 10.1080/14443050802672551

[13] Holt M. Gay men and ambivalence about ‘gay community’: from gay community attachment to personal communities. Culture, Health & Sexuality. 2011;13(8):857–71. 10.1080/13691058.2011.581390 21644116

[14] Davis M, Hart G, Bolding G, Sherr L, Elford J. Sex and the Internet: Gay men, risk reduction and serostatus. Culture, Health & Sexuality. 2006;8(2):161–74. 10.1080/13691050500526126 16641064

[15] Davis M, Hart G, Bolding G, Sherr L, Elford J. E-dating, identity and HIV prevention: Theorising sexualities, risk and network society. Sociology of Health & Illness. 2006;28(4):457–78. 10.1111/j.1467-9566.2006.00501.x 16669808

[16] Frankis J, Oakland J, Lorimer K, Davis M, Flowers P. Social media, Lanarkshire men who have sex with men and sexual health: An experiential qualitative analysis Glasgow: Glasgow Caledonian University, 2014.

[17] Brown M. Gender and sexuality II: There goes the gayborhood? Progress in Human Geography. 2014;38(3):457–65. 10.1177/0309132513484215

[18] Frankis J, Flowers P, Lorimer K, Davis M. Social media, men who have sex with men and sexual health in Lanarkshire Glasgow: Glasgow Caledonian University, 2013.

[19] Zablotska IB, Frankland A, Holt M, de Wit J, Brown G, Maycock B, et al Methodological challenges in collecting social and behavioural data regarding the HIV epidemic among gay and other men who have sex with men in Australia. PLoS ONE. 2014;9(11):e113167 10.1371/journal.pone.0113167 25409440 PMC4237373

[20] Kessel AS, Datta J, Wellings K, Perman S. The ethics of unlinked anonymous testing of blood: views from in-depth interviews with key informants in four countries. BMJ Open. 2012;2(6):e001427 10.1136/bmjopen-2012-001427 . PMC3532979 23263019

[21] Maher D. The ethics of feedback of HIV test results in population-based surveys of HIV infection. Bulletin of the World Health Organization. 2013;91(12):950–6. 10.2471/BLT.13.117309 24347734 PMC3845269

[22] Rennie S, Turner AN, Mupenda B, Behets F. Conducting unlinked anonymous HIV surveillance in developing countries: Ethical, epidemiological, and public health concerns. PLoS Med. 2009;6(1):e1000004 10.1371/journal.pmed.1000004 PMC2628398 19166264

[23] McDaid LM, Hart GJ. Willingness to participate in future HIV prevention studies among gay and bisexual men in Scotland, UK: a challenge for intervention trials. AIDS Behav. 2012;16(6):1420–9. Epub 2011/11/22. 10.1007/s10461-011-0044-6 22101849 PMC3401294

[24] Gammelgaard A, Bisgaard H. Seven-year-old children's perceptions of participating in a comprehensive clinical birth cohort study. Clinical Ethics. 2009;4(2):79–84. 10.1258/ce.2009.009004

[25] Brannen J. Research notes: The effects of research on participants: findings from a study of mothers and employment. The Sociological Review. 1993;41(2):328–46. 10.1111/j.1467-954X.1993.tb00068.x

[26] Lowes L, Gill P. Participants’ experiences of being interviewed about an emotive topic. Journal of Advanced Nursing. 2006;55(5):587–95. 10.1111/j.1365-2648.2006.03950.x 16907790

[27] Parsons S. Understanding participation: Being part of the 1958 National Child Development Study from birth to age 50 London: Centre for Longitudinal Studies, University of London, 2010.

[28] Hunter J, Corcoran K, Leeder S, Phelps K. Appealing to altruism is not enough: Motivators for participating in health services research. Journal of Empirical Research on Human Research Ethics. 2012;7(3):84–90. 10.1525/jer.2012.7.3.84 22850146

[29] Lemke AA, Wolf WA, Hebert-Beirne J, Smith ME. Public and Biobank Participant Attitudes toward Genetic Research Participation and Data Sharing. Public Health Genomics. 2010;13(6):368–77. 10.1159/000276767 . 20805700 PMC2951726

[30] Dixon-Woods M, Tarrant C. Why do people cooperate with medical research? Findings from three studies. Social Science & Medicine. 2009;68(12):2215–22. doi: 10.1016/j.socscimed.2009.03.034. 19394741

[31] Peel E, Parry O, Douglas M, Lawton J. “It’s no skin off my nose”: Why people take part in qualitative research. Qualitative Health Research. 2006;16(10):1335–49. 10.1177/1049732306294511 17079797

[32] Baker L, Lavender T, Tincello D. Factors that influence women's decisions about whether to participate in research: An exploratory study. Birth. 2005;32(1):60–6. 10.1111/j.0730-7659.2005.00346.x 15725206

[33] Townsend A, Cox SM. Accessing health services through the back door: A qualitative interview study investigating reasons why people participate in health research in Canada. BMC medical ethics. 2013;14(40). 10.1186/1472-6939-14-40 PMC3853104 24119203

[34] Hallowell N, Cooke S, Crawford G, Lucassen A, Parker M, Snowdon C. An investigation of patients’ motivations for their participation in genetics-related research. Journal of Medical Ethics. 2010;36(1):37–45. 10.1136/jme.2009.029264 20026692

[35] Sikweyiya Y, Jewkes R. Potential motivations for and perceived risks in research participation: Ethics in health research. Qualitative Health Research. 2013;23(7):999–1009. 10.1177/1049732313490076 23660499

[36] Ritchie J, Spencer L, O'Connor W. Carrying out qualitative analysis In: Ritchie J, Lewis J, editors. Qualitative research practice: A guide for social science students and researchers. London: Sage; 2003 p. 219–62.

[37] White C, Woodfield K, Ritchie J. Reporting and presenting qualitative data In: Ritchie J, Lewis J, editors. Qualitative research practice: A guide for social science students and researchers London: Sage; 2003 p. 287–320.

[38] Anderson B. Imagined communities: Reflections on the origins and spread of nationalism London: Verso; 1983.

[39] Woolwine D. Community in gay male experience and moral discourse. Journal of Homosexuality. 2000;38(4):5–37. 10.1300/J082v38n04_02 10807027

[40] George S, Duran N, Norris K. A Systematic Review of Barriers and Facilitators to Minority Research Participation Among African Americans, Latinos, Asian Americans, and Pacific Islanders. American journal of public health. 2014;104(2):e16–e31. 10.2105/ajph.2013.301706 . PMC3935672 24328648

[41] Fraser S. Getting out in the “real world”: Young men, queer and theories of gay community. Journal of Homosexuality. 2008;55(2):245–64. 10.1080/00918360802129519 18982572

[42] Fraser S. Changing community, changing practice?: Young gay men, HIV, and gay community National Center in HIV Social Research, Faculty of Arts and Social Sciences, University of New South Wales, 2004.

[43] Patterson C, McDaid LM, Hilton S. Gay and bisexual men’s perceptions of the donation and use of human biological samples for research: A qualitative study. PLoS ONE. 2015;10(6):e0129924 10.1371/journal.pone.0129924 . 26053741 PMC4459996

