# Supplementary material for: Understandings of Participation in Behavioural Research: A Qualitative Study of Gay and Bisexual Men in Scotland
Source: PLoS One. 2015 Aug 7;10(8):e0135001. doi: 10.1371/journal.pone.0135001 (PMC4529083; doi:10.1371/journal.pone.0135001)
Supplement: S1 Table — (PDF) [file pone.0135001.s001.pdf]

S1 Table. Participant Characteristics

<table>
<tr><th>Participation</th><th>Pseudonym</th><th>Age group</th><th>Recruitment Location</th></tr>
<tr><td rowspan="19">Survey and sample</td><td>Derek</td><td rowspan="2">unknown</td><td rowspan="2">Glasgow</td></tr>
<tr><td>Nathan</td></tr>
<tr><td>Keith</td><td rowspan="4">18-24</td><td>Glasgow</td></tr>
<tr><td>Lewis</td><td rowspan="3">Edinburgh</td></tr>
<tr><td>Ross</td></tr>
<tr><td>Tomas</td></tr>
<tr><td>Blair</td><td rowspan="5">25-34</td><td rowspan="4">Glasgow</td></tr>
<tr><td>Callum</td></tr>
<tr><td>David</td></tr>
<tr><td>Simon</td></tr>
<tr><td>Ollie</td><td>Edinburgh</td></tr>
<tr><td>Roland</td><td rowspan="3">35-44</td><td rowspan="3">Edinburgh</td></tr>
<tr><td>Hamish</td></tr>
<tr><td>Kennedy</td></tr>
<tr><td>Hector</td><td rowspan="5">45+</td><td rowspan="3">Glasgow</td></tr>
<tr><td>Henry</td></tr>
<tr><td>Taylor</td></tr>
<tr><td>Ben</td><td rowspan="2">Edinburgh</td></tr>
<tr><td>Norman</td></tr>
<tr><td rowspan="10">Survey only</td><td>Harry</td><td>Unknown</td><td>Edinburgh</td></tr>
<tr><td>Bruce</td><td rowspan="2">18-24</td><td rowspan="2">Glasgow</td></tr>
<tr><td>Edward</td></tr>
<tr><td>Cameron</td><td rowspan="2">25-34</td><td rowspan="2">Glasgow</td></tr>
<tr><td>Daniel</td></tr>
<tr><td>Homer</td><td rowspan="3">35-44</td><td>Glasgow</td></tr>
<tr><td>Brodie</td><td rowspan="2">Edinburgh</td></tr>
<tr><td>Roger</td></tr>
<tr><td>Kiram</td><td rowspan="2">45+</td><td>Glasgow</td></tr>
<tr><td>Edgar</td><td>Edinburgh</td></tr>
</table>
